# A current affair: worker perceptions of noise exposure and occupational hearing loss in Australian coal mines

**DOI:** 10.1093/annweh/wxad055

**Published:** 2023-09-16

**Authors:** Adelle Liebenberg, Jacques Oosthuizen, Sue Reed

**Affiliations:** Public Health and Occupational Health & Safety, School of Medical and Health Sciences, Edith Cowan University, 270 Joondalup Drive, Joondalup, WA 6027, Australia; Public Health and Occupational Health & Safety, School of Medical and Health Sciences, Edith Cowan University, 270 Joondalup Drive, Joondalup, WA 6027, Australia; Public Health and Occupational Health & Safety, School of Medical and Health Sciences, Edith Cowan University, 270 Joondalup Drive, Joondalup, WA 6027, Australia

**Keywords:** mining, noise, noise control, occupational hearing loss, worker perceptions, worker beliefs

## Abstract

**Background:**

The objective of the online survey was to determine worker attitudes towards, perceptions on hearing loss, and management of workplace noise; and to identify barriers within current strategies that prevent effective management of hearing health in Australian mines.

**Design:**

This cross-sectional study utilized a modified survey design, initially designed for use by Safe Work Australia for a broader study published in 2010.

**Study Sample:**

The survey questionnaire was made available online to volunteer participants, recruited with the assistance of State and National Health and Safety, and mining organizations. Volunteer participants were required to be proficient in English, be employed by an Australian underground or open cut mine, including coal processing plants; or work as a contractor on one of the specified mine sites. All mining employees, regardless of occupation, job title, and occupational hearing loss classification or status, were invited to complete the questionnaire.

**Results:**

Almost 60% of respondents indicated that they had high noise exposure for than 10 yr or more, and have some trouble hearing, mostly associated with infrequent tinnitus. Nearly 71% of these workers believe that the noise control strategies in their workplaces are effective, but this mostly refers to the use of hearing protection devices.

**Conclusion:**

The results indicate that general knowledge on the cause and effect of noise exposure in the workplace is well understood. However, due to the long latency associated with the development of noise-induced hearing loss (NIHL), there is an issue urgency in terms of risk management. It is surprising that most of the respondents recommended more inspections and administrative controls, especially since most respondents were health, safety, and environment (HSE) professionals. HSE professionals should be advocating for higher order, more permanent solutions, and not purely administrative controls and personal protective equipment. These findings raise the question of whether there is a multifaceted working-culture issue that needs to be addressed, in combination with higher order control implementation.

**What’s Important About This Paper?**

Australian mining industry workers and health, safety, and environment (HSE) professionals surveyed in this study believe noise exposures are well controlled, but hearing protection devices were the primary control strategy used. HSE professionals identified audits, inspections and legislation as the way to solve occupational noise exposure and hearing loss. Given that hearing loss persists in this industry, this study identified the need to increase utilization of engineering control strategies to prevent noise-induced hearing loss.

## Introduction

Global research provides evidence that advocates for the multifaceted benefits of preventing disease, particularly the development of occupational hearing loss (OHL). Preventing hearing loss from progressing reduces the economic burden on industry, society, and the government, and increases worker productivity and wellbeing (Themann and Masterson 2019; Si et al, 2020; Ademi et al. 2021; Zhou et al. 2021). Despite all this research, OHL remains one of the largest compensable occupational diseases across the globe.

Liebenberg et al. (2023) summarized the legal approaches of different countries towards OHL, and where most countries employ a prescriptive approach to the management of workplace health hazards, Australia takes a risk-based management approach. The national model Code of Practice for managing noise and preventing hearing loss at work (Safe Work Australia 2020) places a duty of care on both the employer and the employee to manage and reduce their noise exposure risk in the workplace. Where hazardous noise (>85 dB(A)) is identified as a risk, the employer is responsible to take immediate action to control noise as far as reasonably practicable, and in turn, the employee is responsible to observe and comply with these control strategies advised by the employer (Safe Work Australia 2020). The Code of Practice further states that an employer must review control measures when it is evident that the control measure does not effectively reduce the risk, and where circumstances indicate the health and safety of a member of the work group is affected (Safe Work Australia 2020). The model Code of Practice states that where a worker is identified as having “*sufficient hearing loss to interfere with the safe performance of their work, all reasonably practicable steps should be taken to modify the work environment*” (Safe Work Australia 2020). Thus, where OHL is evident, whether early in its onset or at a more advanced stage, it is the duty of care of the employer and the employee to minimise further risk.

Workplace Health and Safety (WHS) legislation across all Australian States require workplaces to manage and control exposure to excessive noise (>85 dB(A)), following a risk-based approach. Each workplace develops site-specific strategies for meeting these legal obligations, which should be consistent with the anticipated risk of harm to workers. Legislative requirements may involve noise exposure assessment and management and audiometric testing of workers as part of the hearing conservation strategies (Safe Work Australia 2020; Model Work Health and Safety Act, 2022 ), usually forming part of a larger Hearing Conservation Program on site.

Requirements for noise control and audiometric testing has been in place since the late 1970s (Standards Association of Australia., 1976; York, 1980). Current practice for workers who are potentially exposed to noise levels above the exposure standard of 85 dB(A), and where hearing protection devices (HPDs) are required, is that these workers are required to undergo audiometric testing within 3 mo of commencing work, and at least every 2 yr thereafter. More frequent testing may also be required for noise exposures above 100 dB(A) and forms part of the workers’ occupational medical assessment (Safe Work Australia., 2020).

Nevertheless, there is limited research available on site-specific management strategies and how workers with current OHL are identified and accommodated in the workplace. Particularly, how their risk of further hearing loss is mitigated. This research aimed to investigate the strategies implemented for managing and preventing OHL in Australian mining, to enable comparison with the Safe Work Australia (2010) study of other known noisy industries. The research gathered explorative data on the current workplace management strategies for protecting workers with OHL.

The objective of the survey was to determine worker attitudes towards, and perceptions on hearing loss and management of workplace noise (regardless of their hearing status); and to identify barriers within current strategies that prevent efficient and effective management of hearing health in Australian mines.

## Materials and methods

An online survey of workers in the Australian mining industry was undertaken to determine the barriers and beliefs held towards noise control and hearing loss. The objective was to determine the type of noise controls used in mining, and whether HPDs were provided and used at work, including reasons for not wearing HPDs, if there were indications that it is not being used. The survey will also assist the researchers to understand attitudes towards hearing loss and workplace noise.

Workers aged 18 yr and older were asked to complete a 15-min online survey. The survey was available to respondents over 12 mo between 30 January 2022 and 1 February 2023. Ethics approval for this project was obtained from the University of Newcastle Human Ethics Research Committee (H-2022-0047) and Edith Cowan Human Research Ethics Committee (2023-04237-Liebenberg).

### Study design

This cross-sectional study utilized a validated survey, initially designed for use by Safe Work Australia for a broader study published in 2010 (Safe Work Australia 2010). Approval to use the survey was obtained from Safe Work Australia and the original survey questionnaire was provided by the Sweeney Research Institute. It should be noted that approval to use the survey questionnaire does not indicate Safe Work Australia endorsement for the current research. The survey questionnaire was slightly modified to ensure mining-specific job categories and tasks were captured and made available online to volunteer participants. Volunteer participants were required to be proficient in the English language, employed by an Australian mine or processing plant; or work as a contractor on one of the specified mine sites. All mining employees, regardless of occupation, job title, and OHL classification or status, were invited to complete the questionnaire. QuestionPro software (QuestionPro 2022) was utilized for the survey due to the versatility of the software and the associated data security.

### Recruitment

The recruitment of workers was facilitated by Coal Services (NSW), Resources Safety & Health (QLD), Chamber of Minerals and Energy, Australian Institute of Occupational Hygienists, Australian Institute of Health and Safety, and the national Construction, Forestry, Mining and Energy Union. These organizations were asked to distribute the invitations to participate, including the survey link, in their periodical newsletters to the mining industry. Additionally, online platforms such as LinkedIn, Facebook, and Twitter were utilized to post the invitation for workers to participate in an attempt to recruit more workers from a range of the Australian mining industries. Before workers completed the anonymous survey, they were asked to read the Participant Information Statement, acceptance of which implied consent. Participants were asked to provide their contact details, if they were interested in receiving feedback, however these details were not linked to their survey responses in any way.

### Statistical analysis

The online survey data were descriptively analysed. The minimum sample size for the survey was calculated based on the Cochran formula, using the number of workers employed in Australian mining at the commencement of the survey period, *n* = 55,000, (Australian Bureau of Statistics 2017). The number of worker responses required to allow a 95% confidence level, with a 10% margin of error in the validity of the results for a population of *n* = 55,000, was 96 participants.

## Results

The survey was accessed a total of 946 times, 72 workers responded to the questionnaire, at least in part, and 48 (66.7%) completed the entire survey. As not all workers completed all the questions, the response rate for each question is indicated for each of the result categories. The number of respondents (*n* = 72) were lower than the number required for 95% confidence level in the validity of the results, however, this does meet the minimum number of respondents for a 90% confidence level in the results (*n* = 68). Due to the low confidence level of the results, these results cannot be generalized across the Australian mining population. The data does, however, provide a valuable insight into the perceptions and beliefs of these workers regarding noise control in the mining sector.

### Demographics

Table 1 summarizes the demographic profile of the respondents, which shows the majority were female (73%), aged 25 yr and above. Approximately 44% of respondents were in the age range of 35–49 yr. The survey responses were provided by workers from New South Wales (26%), Queensland (36%) and Western Australia (34%) primarily. There were no respondents from the Australian Capital Territory nor South Australia (SA).

**Table 1.** Demographical composition of perceptions and beliefs of OHL survey respondents.

Variable	Number of responses (*N*)	% of *N*) population
Sex	56	
Male	14	25
Female	41	73
Prefer not to say	1	2
Age (years)	62	
20–29 yr	4	6
30–39 yr	13	21
40–49 yr	19	31
50–59 yr	20	32
60+ yr	6	10
State	61	
Australian Capital Territory (ACT)	0	0
New South Wales (NSW)	16	26
Northern Territory (NT)	0	0
Queensland (QLD)	22	36
South Australia (SA)	0	0
Tasmania (TAS)	1	2
Victoria (VIC)	1	2
Western Australia (WA)	21	34

Workers employed at underground (30%) and open cut mines (36%), or a combination of these (25%) were the primary respondents to the survey (Table 2). It is likely that the combination of the underground and open-cut mine employment category includes consultants and contractors who were undertaking work on different mine sites. It is interesting to note that there were no respondents from the development crews or surveyors (non-production sections of the mines). Health, safety, and environmental (HSE) officers were the largest group of respondents (36%), followed by production workers (16%) and maintenance workers (11%).

**Table 2.** Summary of mine classification, employment, and job title of respondents.

Variable	Number of responses (*N*)	% of population
Mine classification	61	
Open cut	22	36
Underground	18	30
Processing plant	1	2
Combination	15	25
Other (Consultant/Contractor)	5	8
Main section	61	
Production	10	16
Maintenance	6	10
Development	0	0
Shot firers/ Blast crew	3	5
Surveyors	0	0
Processing plant	1	2
Health, safety and environment	30	49
Roving (Admin, Office, Utilities, Managers)	7	11
Consultant/ Contractor	4	7
Role descriptor	64	
Administrative staff	3	5
Building/ Engineering Technician	1	2
Consultant/ Contractor	6	9
Health, safety and environment officer	23	36
Heavy mobile equipment Operator	3	5
Inspector	5	8
Maintenance crew	3	5
Manager/Superintendent (All)	6	9
Mine Engineer/Engineering professional	2	3
Production crew	10	16
Supervisor	2	3

### Noise exposure: awareness, causes, and consequences

Table 3 shows that most of the respondents had worked in loud noise for more than 10 yr (58%), and with fewer working between 1 and 10 yr (20%). Eighty percent of respondents indicated that they are normally exposed to loud noise multiple times a day, for up to 5 h.

**Table 3.** Self-reported noise exposure of respondents.

Variable	Number of responses (*N*)	% of population
How long have you worked in loud noise	60	
Never worked in loud noise	3	5
<1 yr	7	12
1–10 yr	12	20
>10 yr	35	58
Unsure	3	5
How often do you work in loud noise (last 2 wk)	61	
About 1–2 periods	27	44
Several loud periods a day	22	36
Constant exposure all day	6	10
Unsure	6	10
What was your level of noise exposure the last 2 wk?	53	
About the same	44	83
More than usual	6	11
Less than usual	2	4
Don’t know	1	2
How often do you work in loud noise/shift?	61	
None	8	13
<2 h	22	36
2–5 h	16	26
6–10 h	8	13
<10 h	7	11

Eight of the respondents indicated that they were not exposed to loud noise in the 2 wk prior to completing the survey due to not being exposed to loud noise in their current role (*n* = 6), and their current workplace did not typically produce loud noise (*n* = 2).

The respondents indicated that most of the noise (54%) was generated using various mining machinery. Continuous running machinery and iron and ore crushers (28%), tools such as power tools, air tools, compressors (38%), and air conditioning and exhaust fans (18%) are considered the main contributors to overall noise exposure profiles. The second largest noise contributor was believed to be vehicles and transport (33%), which was stratified into categories as follows heavy mobile equipment (41%), light vehicles (29%), reversing alarms (28%), and aircraft (2%). Specific activities such as blasting and welding or steel cutting contributed to 7% (*n* = 16) of the perceived noise exposure profile, followed by loudspeakers, radios, or music (6%).

Forty-two percent of respondents believed they have good hearing (42%), whereas 48% indicated they have some trouble hearing, and 7% reported a lot of trouble hearing. Three percent of respondents were unsure how to describe their hearing when not wearing HPDs. Most respondents (31%) indicated they were worried about their hearing while working in loud noise, whereas 26% indicated that working in loud noise makes them feel irritated, 16% indicated that they were less enthusiastic about work or tired (11%), due to the noise levels, while 13% of respondents indicated that working in loud noise did not bother them.

A quarter of respondents indicated that they had not considered the prospect of losing their hearing, while 67% had thought about hearing loss. When asked if they have experienced tinnitus, or a ringing in their ears, 23% of the respondents indicated that they experience tinnitus constantly, while 48% experience tinnitus sometimes, and 23% had never experienced it. Fifty-nine percent of respondents indicated they believe they are more careful than most of their work mates in protecting their hearing, and 31% indicated that they were similar to their colleagues regarding hearing loss and management. The remaining 10% indicated that they do not believe they are as careful as their work mates.

**Figure 1:**
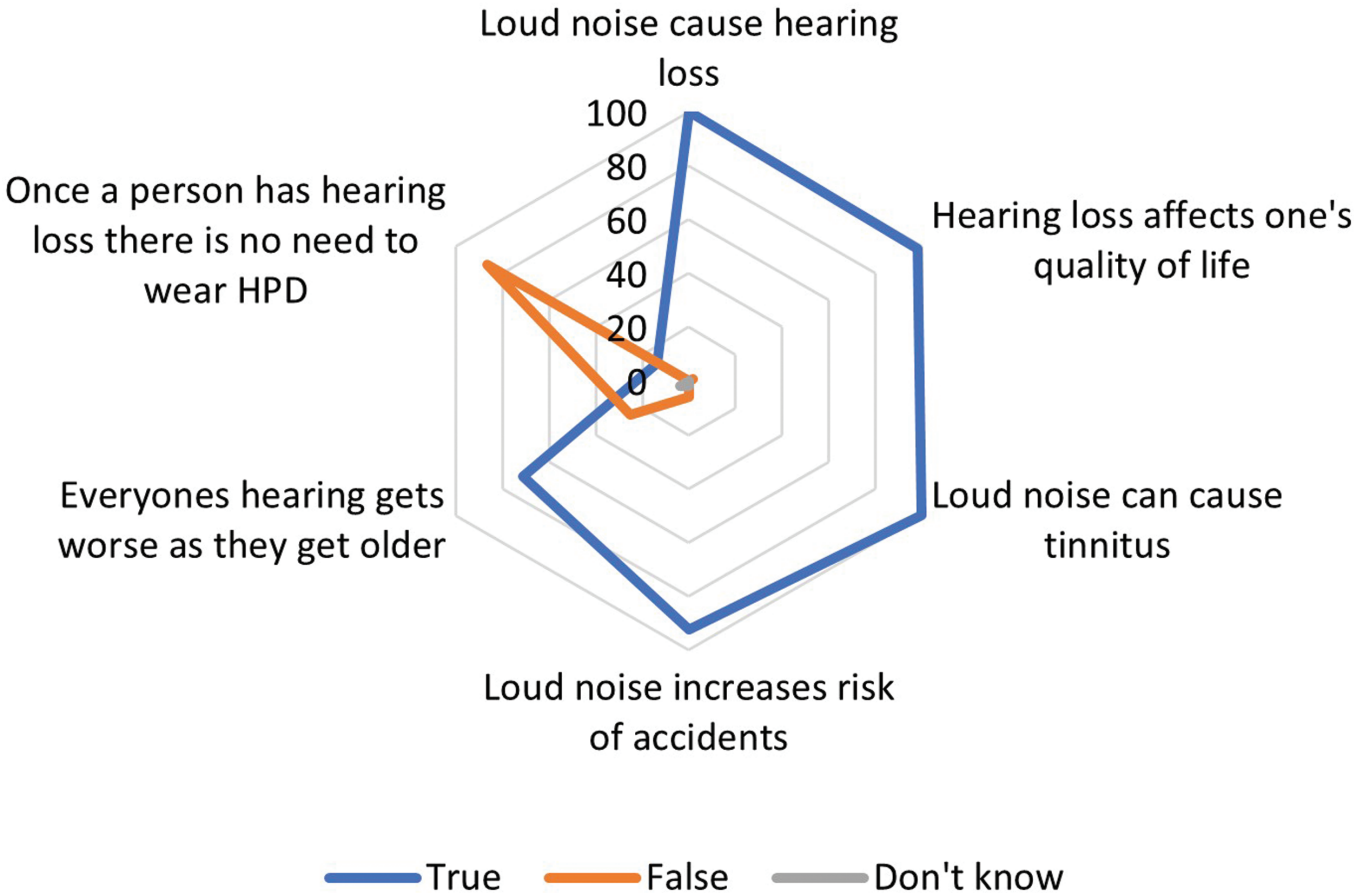
Worker Perceptions on Hearing Loss.

When assessing the worker beliefs regarding hearing loss (HL), all respondents believe that loud noise can cause HL and tinnitus (Fig. 1). Most workers also indicated that they believe that HL affects the quality of one’s life, and that loud noise can increase the risk of accidents (98% and 92%, respectively). Contrary to expectation, 25% of respondents did not believe that age-related HL occurs as a person ages, and 13.5% believe that HPDs are not required once HL is present.

### Noise control: implementation and efficiency

The results of the survey indicate that workers believe noise sources are well controlled in their workplace; however, ~90% of all controls reported were limited to the provision of HPDs. Additionally, most of the participants in the study indicated that management of noise was not viewed as a significant health concern in their workplace and was largely ignored, similar to the findings of Safe Work Australia more than a decade ago (Safe Work Australia 2010).

Total elimination of noise generation in mining is unlikely due to the nature of the industry which involves crushing of rock and ore, thus preventing the total elimination of noise as a primary control. The controls used throughout the Australian mining industry, as identified through the survey are summarized in Table 4. Administrative controls (55%) and the supply of personal protective equipment (PPE), specifically HPDs (29%), are the primary controls identified, which highlights the lack of compliance with the legislative approach of the last 40 plus years.

**Table 4.** Type of noise controls implemented in Australian mining.

Controls	Responses (*N*)
Isolation/Substitution	22 (5%)
Noise isolated	12
Sound absorbing material	10
Barriers between sources and workers	10
Engineering	17 (4%)
Modifications to machines	10
Administrative	241 (55%)
Audits/Inspections	27
Audiometric testing	39
Buy quiet policy	10
Compliance with Government Standards	20
Employees informed about Health and Safety	25
Internal company guidelines	23
Job rotation	6
Scheduling/ Rotation of work allocation	5
Training	30
Warning/ Advice to keep clear	26
Noise exposure monitoring	30
PPE	125 (29%)
Earmuffs provided	42
Ear plugs provided	46
HPD fit testing	27
Other PPE	10
None	31 (7%)

Respondents (*n* = 60) indicated that their workplaces provide HPDs, and 85% of these workers always wear their HPD as required. The primary reasons for not wearing HPD in loud noise is that having a conversation is hard (46%), and that they do not feel that they are exposed to the loud noise for long enough to warrant wearing HPD (30%). Other reasons include discomfort whilst wearing HPDs (11%), not being able to hear warning signals (5%), the belief that HPD causes ear infections (5%), and that they are unsure how to obtain a good fit or seal when using HPDs (33%).

**Figure 2:**
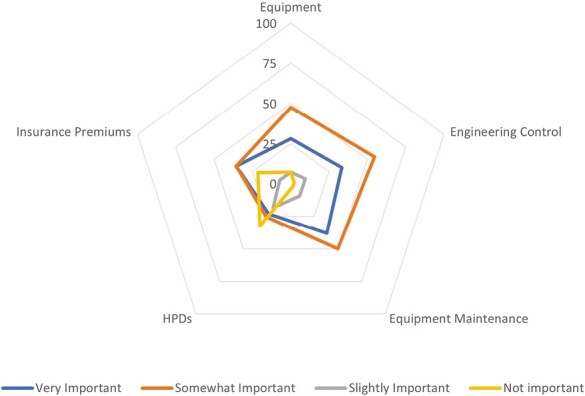
Importance of cost when considering noise controls.

Respondents indicated the cost of equipment maintenance (38%), and insurance premiums (36%) were considered particularly important when considering noise control in the workplace, with engineering control costs (55%) and equipment cost (48%) considered somewhat important. Surprisingly, the cost of HPDs were not considered important (33%) with regard to noise control (Fig. 2).

Half of the respondents (50%) indicated that noise is somewhat controlled in their workplace but also felt that some health and safety rules are not practical and that accidents are likely to occur regardless of prevention strategies implemented. With relation to noise control, the respondents believe that the added benefits of effective noise control include improved worker morale (39%), resulted in fewer compensation claims (34%), and increased productivity (17%). Very few (5%) believe that effective noise control reduces accidents in the workplace.

The priorities of noise control and beliefs highlighted by the respondents include that hearing loss in mining is an important issue (11%) for which more education and awareness training is required (11%) and they believe that it is an issue not taken seriously (9%). The respondents also indicated that they believe individuals need to take responsibility (9%) to protect their own hearing.

Furthermore, these workers believe that noise cannot be eliminated from industry and that management needs to do more to protect their workers. There is a belief that some workplaces are negligent, and that workers have suffered hearing loss as a direct result of workplace noise. Workers also believe that changes are needed, such as more and tighter regulations, introducing legislating HPD fit testing. There is also a need to focus on higher order controls regarding noise control.

## Discussion

### Noise exposure: awareness, causes, and consequences

The objective of this study was to investigate the perceptions and beliefs of Australian mine workers regarding noise exposure and hearing loss management. A telephone study undertaken by Safe Work Australia (SWA) in 2010 aimed to establish the barriers and enablers to effective noise control and prevention of OHL. The Safe Work Australia (2010) study targeted 5 industry groups associated with high noise exposure: (i) manufacturing, (ii) construction, (iii) transport and storage, (iv) hospitality and entertainment, and (v) agriculture, forestry, and fishing. No data were collected for mining, an industry renowned for high noise exposure (Bauer and Kohler 2000) and hearing loss, (Liebenberg et al. 2021), hence the focus for this study on mining specifically.

The current research found that general knowledge on the cause and effect of noise exposure in the workplace is well understood, but due to the long latency of noise-induced hearing loss (NIHL), lacked urgency in terms of risk management. Kanji et al. (2019) found that workers employed in the mining industry are generally aware that it is a noisy industry due to the inherent nature of the work undertaken. In contrast, the earlier Safe Work Australia study found a failure to link cause and effect, underpinned by an observed reluctance to adopt preventative action for noise exposure management (Safe Work Australia 2010).

Most workers believe that loud noise cause HL and is also the cause of tinnitus. Whilst this is true for HL, the development of tinnitus is idiopathic. Research indicates that the onset of tinnitus is associated with sensorineural HL, ear injuries, or circulatory issues (Dalrymple et al. 2021); however, the exact mechanism is unknown.

The Safe Work Australia (2010) study found that workers are more worried about developing tinnitus compared to developing OHL. It is concerning that 72% of the respondents indicated they experience some form of tinnitus whether continuously or intermittently.

Almost 60% of respondents indicated that they had high noise exposure for more than 10 yr. This is typically where one would observe workers starting to present with noticeable signs of early onset NIHL. Consequently, it is expected that two of the major aspects highlighted by the worker responses were irritability and concern for their hearing when working in loud noise environments (Basner et al. 2015).

The main sources of noise were believed to be heavy mobile equipment (HME), light vehicles, power tools, air tools and compressors; and mining machinery including crushers and the ventilation systems. These findings align with noise exposure assessments undertaken in the mining industry globally. Studies by Bauer and Kohler (2000) and McBride (2004) found that heavy mobile equipment generates noise levels between 90–102 dB(A), pneumatic percussion tools 114–120 dB(A), cutting machines, conveyers, pumps, and continues miners between 83–102 dB(A), drilling machines and shovels 80–90 dB(A), and fans between 90–110 dB(A). A recent study in the United States investigated the relationship between noise exposure and the risk of injury among mine workers, finding a definitive dose–response relationship between noise exposure and work-related injuries. Where noise exposure exceeded 88 dB(A), a 17.9% increase in work-related injuries were observed (Shkembi et al. 2022). These findings align with previous research on the relationship between noise exposure and risk of injury (Neitzel et al. 2016), highlighting the co-benefit of addressing noise exposure, particularly in mining where the noise exposure levels are typically high.

### Noise control: implementation and efficiency

The current research found that besides lower noise levels, higher worker morale and fewer compensation claims are the benefits of improved noise control benefits that most respondents considered important regarding noise control. Previously, the risk of workers’ compensation for OHL was found not to be an enabler for noise control (Safe Work Australia 2010). Increased productivity and safety were other less commonly perceived benefits of noise control, and the perceived cost of noise control equipment and insurance premiums is seen as a barrier to effective noise control in the workplace.

Only 9% of respondents in this study indicated higher order controls such as isolation and substitution solutions are considered for implementation in mining, with most respondents not aware of any intentional investment by their companies towards noise control. These responses are mirrored by a scoping review completed Liebenberg et al. (in press) that investigated the interventions and controls available to workers with OHL.

Nearly 71% of workers believe that the noise control strategies in their workplaces are effective, but this mostly refers to the use of HPDs. Over reliance on HPDs appears to be as common now as it was more than a decade ago (Safe Work Australia 2010). Twenty-nine percent of controls used related to HPD and HPD fit testing. Personal protective equipment such as HPD are the last line of defence for workers, and the lowest level in the hierarchy of control. If these fail or malfunction, workers are no longer protected against the noise hazard. Respondents indicated they mostly wear their HPDs as required, except when needing to communicate with others, where they were not prepared for, nor expected loud noise in the area, or do not feel they are exposed long enough to cause HL. This indicates that these workers have insufficient knowledge regarding implementing noise control and the implications of not wearing HPD appropriately.

One common issue reported with wearing HPDs is the perceived inability to communicate well. This is usually an indication that the correct type of HPDs have not been selected or are incorrectly used, for the noise levels generated in the areas of exposure. Many workplaces purchase a standard class of HPD, usually class 5, to accommodate for “worst case” scenarios. However, noise generated from mining equipment has been measured to range between 80–120 dB(A), which requires that the correct classes of HPDs are selected, used, and maintained, for different tasks and exposure profiles. This would not only aid in the prevention of noise-induced hearing loss, but it would also allow for effective communication to occur when used appropriately.

When asked about controls employed at their workplace, other than HPDs, the respondents indicated administrative controls (55%) such as audiometric testing and awareness training, audits and inspections, signage, policies, and procedures were common. Hearing loss is believed to be a principal issue, and more education and awareness training is required in addition to undertaking more compliance inspections. The fact that most respondents recommend more inspections and administrative controls is surprising, specifically as most of the respondents self-reported as HSE professionals. Noise control is a complex issue and there are no easy, “one-size fits all” solutions. It should also be noted that complete elimination of noise in the mining sector is unlikely due to the nature of the operations (crushing of rock and ore). Nevertheless, HSE professionals should be advocating for higher order, more permanent solutions rather than lower order, administrative, and personal protective equipment controls. Overall, the respondents reported positive attitudes towards noise control to help prevent hearing loss. However, the results of this survey indicate that the respondents do not engage in frequent and appropriate use of HPDs.

Research indicates that although workers might feel positive towards a certain outcome, they do not always follow this belief with appropriate behaviour and action. Maglio et al. (2016) and Harrison et al. (2018) investigated the barriers and beliefs of firefighters towards the decontamination of firefighter equipment, to prevent the development of cancer, finding that a positive attitude towards a specific issue is not the primary motivator for a behaviour change. These studies have demonstrated that peer influence is an important predictor to the correct use of protective equipment in firefighters. It is likely that similar attitudes towards HPD influence individual behaviour in the mining sector as well. These workers indicate positive attitudes towards prevention and control, but their behaviour does not reflect these attitudes.

This finding raises the question whether there is a deeper working-culture issue that needs to be addressed, instead of undertaking more noise assessments, training, and enforcing legislative requirements. Particularly as most of the respondents believe that some Health and Safety rules and regulations are not practicable (63%), and that regardless of control implementation, accidents are still likely to occur in their workplace (50%).

Future research is needed to determine the psychosocial aspects behind the fatalistic acceptance of noise exposure at work and the development of NIHL and raise the profile of this topic in industry. Secondly, it is recommended that training and awareness campaigns such as Dangerous Decibels (Dangerous Decibels 2019) for younger generations must be implemented to ingrain adequate risk awareness and affect generational change in behaviours. Many large studies have been undertaken to estimate the productivity burden of occupational noise-induced hearing loss (Nelson et al. 2005; Lim et al. 2012; Vos et al. 2012; Si et al. 2020; Ademi et al. 2021; Man et al. 2021). Site-specific cost-benefit analysis of effective noise exposure control should be included as a key element in any hearing conservation program to communicate the genuine cost of inefficient noise control. If these costs were incorporated into the site-specific hearing conservation plan, it would validate the expenses associated with higher-order controls such as engineering and isolation controls, and ultimately reduce hearing loss and ongoing costs.

### Limitations

The number of respondents (*n* = 72) was lower than that required (*n* = 96) to achieve a 95% confidence level in the validity of the results (10% margin of error). The results of the study indicate an over-representation of females (41%) compared to the industry average and are likely to be biased towards workers with an interest in the topic. The results of this study can thus not be generalized across the whole Australian Mining population. The results of the survey still provide valuable insights into the perceptions and beliefs of these workers regarding noise control in the mining sector. Furthermore, many of the respondents (36%) were HSE professionals, which increases self-selection bias of the survey results.

## Conclusion

The results of the current research indicate that although the industry sector is different compared to the original sectors included in the Safe Work Australia (2010) survey, that not much has changed in the beliefs of workers employed in noisy industries. Furthermore, there is still an over-reliance on hearing protection devices as a primary method of controlling noise exposure. Future research should investigate whether a multifaceted approach could be required to address hearing loss as a “whole of life” issue compared to a silo approach between workplace noise exposure and social noise exposure, as workers are exposed to noise through social events and hobbies as well (Smith et al. 2000; Bott and Saunders 2021). One element that should be pursued is educating younger generations on critical risks and behaviours, particularly excessive noise exposure and hearing loss. Similar campaigns have been successful in the fields of recycling (Grodzinska-Jurczak et al. 2003) to raise awareness and change inter-generational behaviour.

## Data Availability

The data underlying this article will be shared on reasonable request to the corresponding author.
